# Motion-numerical compatibility affects magnitude classification

**DOI:** 10.1038/s41598-026-37414-0

**Published:** 2026-02-04

**Authors:** Vittoria Volpi, Carlotta Isabella Zona, Martin H. Fischer

**Affiliations:** 1 University of Paris Cité, Paris, France; 2 University of Potsdam, Potsdam, Germany

**Keywords:** Spatial-numerical association, SNARC, Motion-numerical compatibility, Go/No-go task, Neuroscience, Psychology, Psychology

## Abstract

Number concepts are spatially organized along a mental-number line (MNL) with smaller magnitudes represented in left/lower space and larger magnitudes progressively toward right/upper space. Evidence for this association largely relies on simple button responses. We investigated the generality of this association with directional head movements. In a preliminary test, 31 neurotypical adults classified spoken numbers with lateralized buttons faster under MNL-congruent than incongruent conditions along both horizontal and vertical axes. Having established the standard number-space association, participants then performed a Go/No-go version of our task with concurrent horizontal or vertical head movements. The results showed a congruency effect between number magnitude and head direction—which was limited to the horizontal axis. Our results suggest that horizontal motion induces spatial attention shifts which facilitate spatially congruent magnitude processing, thus supporting a conceptual association of space and magnitude.

## Introduction

Humans often rely on spatial associations to organize and retrieve their knowledge, be it about narrative structures, social relations, or other arbitrary symbolic associations (reviewed in^1^). This spatial-conceptual association also applies to abstract knowledge domains. For example, while numbers and mathematics have been traditionally considered as abstract knowledge domains *par excellence*, extensive evidence demonstrated that the cognitive organization of number knowledge is spatial in nature (reviewed in^2,3^). For example, when individuals (especially Westerners) respond to small numbers like 1 or 2, their response is faster with a button located on their left than with a button on their right side; conversely, when they respond to larger numbers like 8 or 9, their response is faster with a button located on their right than with a button on their left side^2,4^. This *Spatial-Numerical Association of Response Codes* (SNARC) effect obtains with many number formats (auditory, visual, digits and words) and different choice response tasks (e.g., magnitude comparison or classification, parity classification; reviewed in^5^. The widely agreed interpretation of this association is that it reflects a cognitive compatibility of number magnitude with horizontal space, with smaller numbers mentally represented to the left of larger numbers on a *mental number line* (MNL). Using the choice response task, recent studies also reported number-space associations along the vertical axis^6–11^, linking smaller numbers to lower space and larger numbers to upper space. However, early investigations of vertical SNARC effects reported mixed directions of the association, with some studies finding the opposite pattern (e.g^12–14^) suggesting that task demands and contextual factors may modulate how vertical space is mapped onto number magnitude.

Several mechanisms have been proposed to explain the emergence of the SNARC effect. One candidate mechanism was the categorical coding of stimuli and responses as binary pairs with similar (positive or negative) polarity, such as “left” and “small” both being coded as negative poles, and “right” and “large” both being coded as positive poles. Under the *polarity correspondence* hypothesis^15^ concepts with identical poles facilitate cognitive processing. However, recent studies eliminated this contamination of conceptual processing with response coding by using only a single response location. Specifically, go/no-go tasks require no response coding and still detect spatial-numerical associations (hence labelled SNAs; cf.^16,17^; see also^18^). This devalues the explanatory power of the polarity correspondence hypothesis.

Alternatively, SNAs might reflect the use of *linguistic metaphors*, according to which “more” is associated with “up” and “fewer” with “down” (cf. expressions like high vs. low price, high vs. low value, or high vs. low temperature, which exist in most languages). However, this explanation applies only (and even inconsistently) to vertical SNAs, while corresponding metaphorical expressions for the horizontal dimension seem lacking.

Finally, a long-standing hypothesis is that mental space may be isomorphic to physical space, i.e., that the functional relations among cognitive representations parallel those among their external referents^19^. Within this cognitive representation, *conceptual similarity* is thought to be operationalized as spatial distance across items, which are organized along ordered continua that represent relevant dimensions^20^. For example, number concepts are ordered progressively according to their magnitude on the MNL. Recent evidence has supported this view by showing that the brain structures evolved to enable physical navigation are also implicated in the storage and manipulation of abstract knowledge—in other words, in the “navigation” of conceptual spaces, whose cognitive representation is a map of the schematic relationships across landmarks (i.e., concepts) in an environment (the conceptual space, for reviews, see^1,21^).

The systematic deployment of spatial attention to specific areas of space has been proposed as potential mechanism driving facilitation in the processing of numerical magnitudes represented in the same space, both in overt (observable) orienting behavior and as inferred from performance differences when directed covertly. Overt and covert attention are tightly coupled and goal-directed movements are generally preceded by endogenous (i.e., voluntary or intentional) shifts of covert spatial attention towards their goal locations as part of movement planning (for hand movements:^22,23^; for eye movements:^24,25^; for a recent review see^26^).

In support of an attentional mechanism underlying SNAs, unimanual pointing completes faster to left/right targets in response to centrally presented small/large numbers^27^. Similarly, participants spontaneously direct their gaze to magnitude-congruent areas of vertical and horizontal space during counting^28^ and magnitude processing^29^. Other research showed SNAs also in mouse-cursor and finger-pointing trajectories^30,31^, and even when responses were made independent of numerical processing^32^. Together, this evidence suggests that activating number concepts biases motor behavior toward magnitude-congruent regions of space, in a tight interplay of physical movement, visuo-spatial attention, and higher cognitive processing.

Importantly, this attentional interplay between number and movement is bidirectional, such that directional movements also affect number production. Consistent with such bidirectionality, whole-body movements can influence arithmetic performance, facilitating calculations when the movement direction is congruent with the operation (leftward for subtractions, rightward for additions) compared with incongruent movement^33^. In children, full-body movements on a digital dance mat can improve number-line estimation and general arithmetic performance^34^. This suggests that sensorimotor spatial training can enhance the precision of mental number-line representations, reinforcing the notion that number magnitude is grounded in spatial and bodily experience. Consistently, in random-number generation (RNG) tasks, participants tend to generate larger numbers after right compared to left head turns^35^ (see^36,37^, for replication and extension to arm movements and passive motion). Similarly, participants in Shaki and Fischer’s (^38^ Experiment 2) study performed the RNG task during walking and generated smaller numbers preceding an instructed left turn compared to an instructed right turn. These findings indicate that body movements shift attention along a horizontally oriented MNL, making spatially compatible number concepts cognitively more available.

This phenomenon has been labelled *motion-numerical compatibility* (MNC) effect by Cheng et al.^36^: Movements of the body in physical space trigger shifts of spatial attention that, in turn, activate conceptual representations associated with corresponding areas of mental space—in the case of numbers, through their ordered progression on the MNL. However, evidence for the MNC effect so far has been limited both with respect to the tasks and the spatial dimensions under investigation. We briefly consider these aspects to motivate the present study.

As reviewed above, a dominant task so far was RNG, making movement direction the independent variable and number magnitude the dependent variable. An exception is the report by Stoianov et al.^39^, where covert attention was manipulated prior to number classification. Participants were quicker to classify numbers as smaller than a fixed reference when the left-hand side was cued, and vice versa for larger numbers when the right-hand side was cued, signaling the bi-directional link between space and numbers. However, this study included no body-movement manipulation and used lateralized response buttons, thus contaminating the assessment with peripheral response coding. It remains unknown whether an intentional directional movement influences other aspects of numerical cognition, such as the latency with which participants judge (rather than produce) magnitudes.

Consider now the spatial dimensions that were studied in previous work on MNC. As reviewed, most prior research focused on horizontal movements of various body parts. One exception from this is the work of Winter and Matlock^40^, who found SNAs for vertical head movements during RNG. Therefore, it is worth studying whether the MNC effect emerges again when performing vertical head movements concurrently with another task.

Given that large vertical head movements make the visual perception of stimuli at a fixed location challenging (and potentially introduce new spatial biases), we resorted to auditory number presentation, which is rather infrequently used in SNA studies^9,41^. Therefore, our study progressed in two steps: First, we established in a baseline test the presence of horizontal and vertical SNARC effects with spoken stimuli in our participants. Then we investigated whether the same participants’ horizontal and/or vertical head motion affected their processing of those spoken stimuli in a magnitude classification task. We hypothesized that movement-induced shifts of attention toward specific areas of external space, induced by the planning of directional head movements—rather than by the head’s final location—would activate magnitudes represented in the same areas of mental space, thus speeding up their classification. Specifically, leftward and downward head motion should cue attention towards left and lower space, respectively. This should in turn facilitate the classification of smaller magnitudes. Conversely, rightward and upward motion should direct attention toward right and upper space, respectively, thereby facilitating the classification of larger magnitudes. The present work therefore contributes to ongoing debates in numerical cognition by integrating evidence from spatial attention, embodiment, and motion. Our paradigm, without relying on bilateral response mappings, examines how planning body movements, in both the horizontal and vertical axes, influences the processing of numerical magnitude itself, rather than its production. By examining how active body movements modulate magnitude processing, we aim to clarify how spatial and sensorimotor factors shape abstract numerical representations.

## Results

**Fig. 1:**
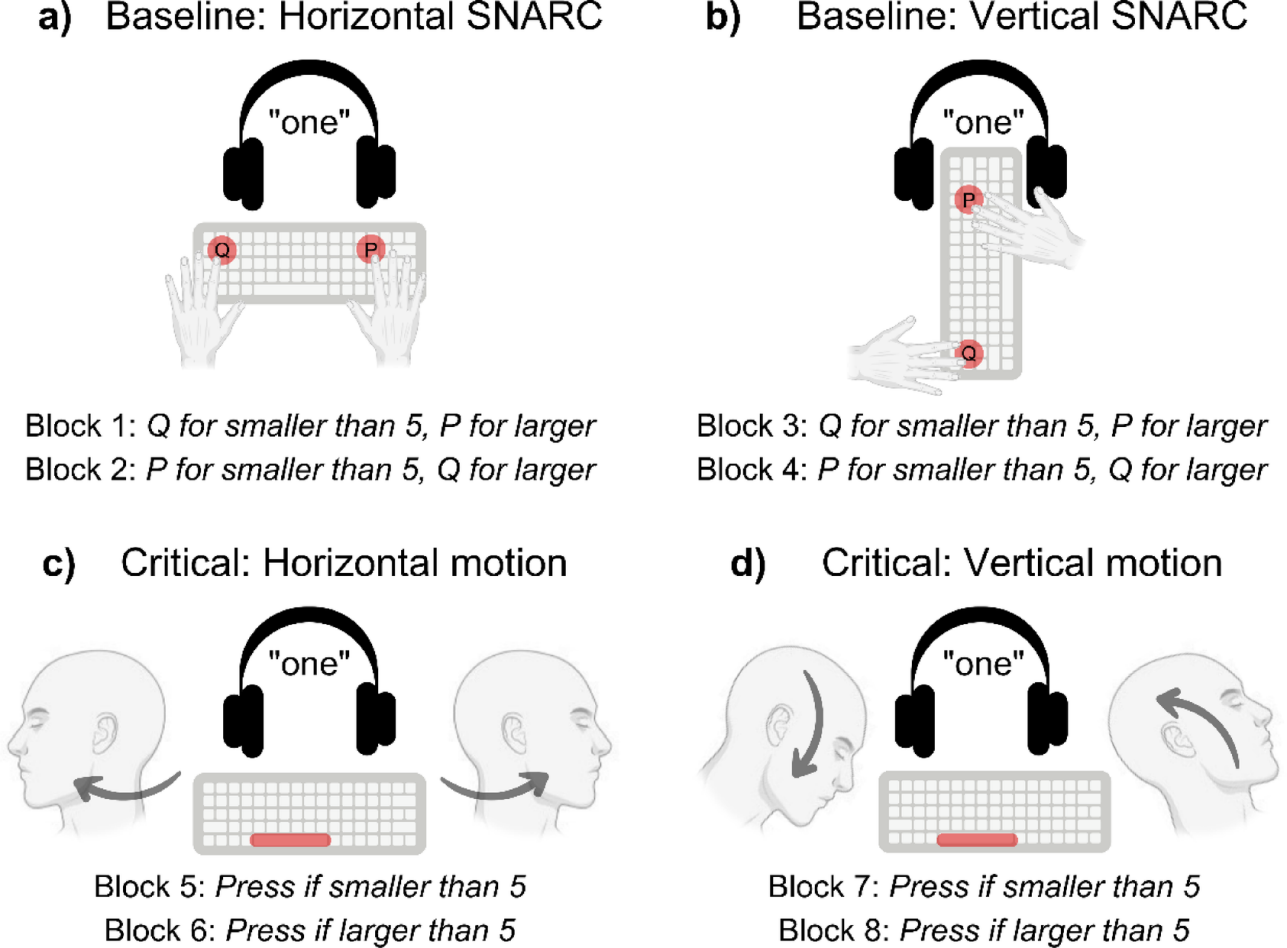
Experimental Blocks and Instructions. Note that the top-left panel shows the horizontal condition from the participant’s perspective, while the top-right panel shows the vertical condition from a frontal, external view, as in the vertical blocks the keyboard was held upright facing away from the participant.

We studied performance under two task instructions. In a baseline test, we documented a typical SNARC effect along horizontal and vertical dimensions in a magnitude-classification task with spoken number stimuli and two response buttons (baseline “SNARC” blocks; top panels of Fig. 1). This task served to validate our stimulus materials for the main experiment. For the critical blocks, we asked the same participants to perform repetitive horizontal or vertical head movements to induce endogenous attention shifts; each time a spoken number was presented, they classified it as larger/smaller than five by pressing the spacebar under different Go/No-go instructions (bottom panels of Fig. 1) prior to completing the planned head rotation. Thus, each magnitude-classification response was provided directly before a directional head movement, and the outcomes were analyzed as a function of this following movement.

### Descriptives

**Fig. 2:**
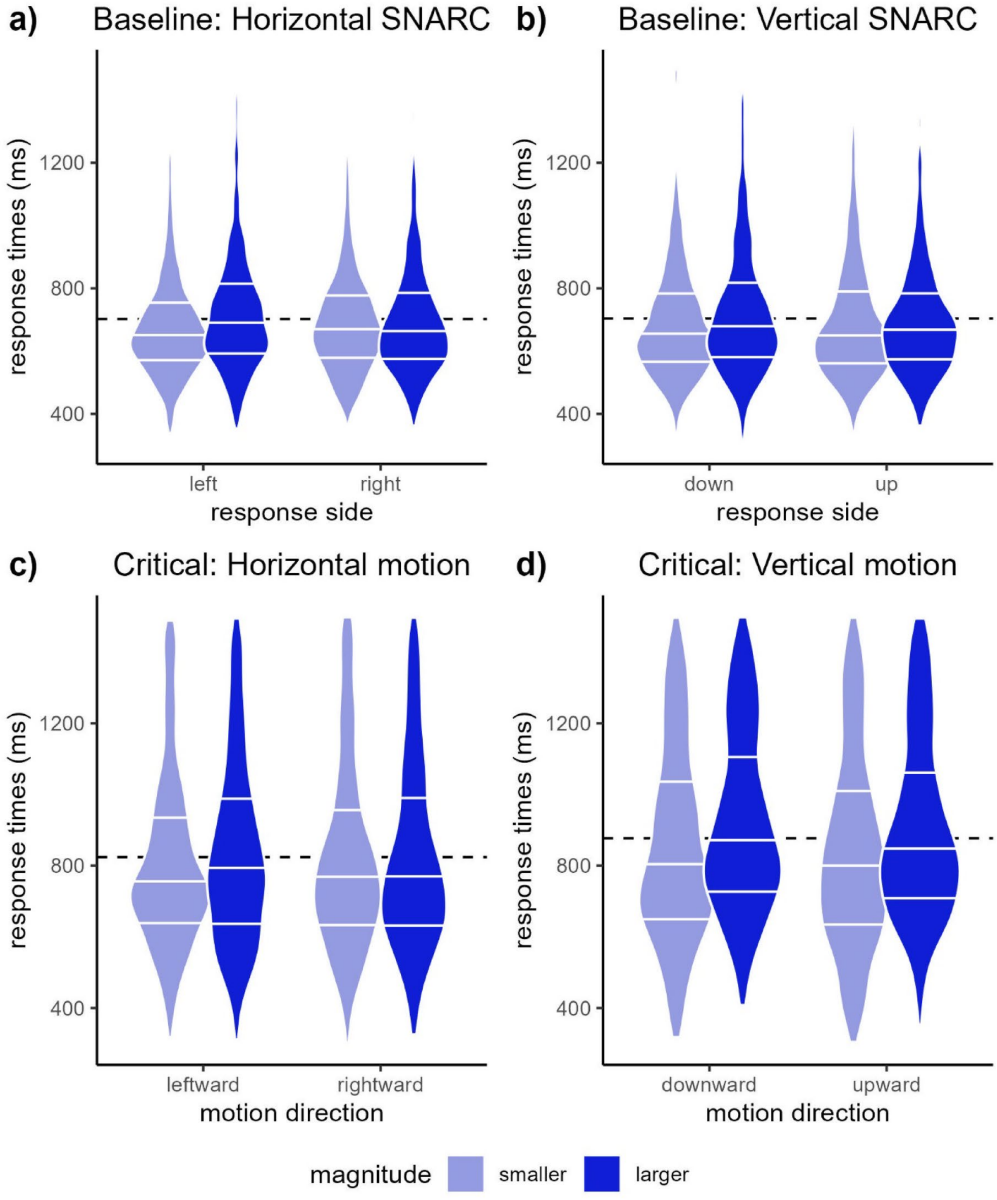
Average Response Times in each Condition. Horizontal white bars correspond to 25th, 50th, and 75th percentiles of the distribution. Dotted lines indicate mean RTs across conditions within each Block.

Average by-participant response times (RTs) in each block (correctly answered and Go trials only) are illustrated in Fig. 2 as a function of number magnitude and response side (Baseline Blocks: upper panels) and head-motion direction (Critical Blocks: lower panels).

### Baseline blocks: evidence for horizontal and vertical SNARC effects

Table 1 reports the estimates from the mixed-effects linear regression models fitted to natural-log transformed RT data from horizontal and vertical baseline SNARC conditions. The accuracy percentage was 97.47% (SD = 16.70%) with congruent (i.e., left-small and right-large) response-button mappings, and 95.83% (SD = 19.99%) with incongruent mappings.

**Table 1:** Output of mixed-effects linear regression from baseline SNARC Blocks.

Predictor	Horizontal SNARC				Vertical SNARC			
	Est.	SE	t value	*p* value	Est.	SE	t value	*p* value
Intercept	6.52	0.03	193.51	< 0.001	6.53	0.03	202.14	< 0.001
Magnitude	− 0.03	0.05	− 0.65	0.582	− 0.02	0.04	− 0.59	0.615
Response Side	< 0.01	0.01	− 0.68	0.498	− 0.02	0.01	− 2.41	**0.016**
Magnitude × Response Side	0.04	0.01	2.62	**0.008**	0.03	0.01	2.15	**0.031**

Note Formula: log(RT) ~ Magnitude * Response Side + (Magnitude | Participant) + (1 | Number).

Significant values are in bold.

In the vertical SNARC condition, responses given with the top key tended to be associated with shorter RTs compared to responses given with the bottom key (effect of Response Side, marginal).

Importantly, the models revealed SNARC effects in both spatial dimensions, indexed by significant interactions of Magnitude × Response Side (horizontal: *f*^*2*^ = 0.22; vertical: *f*^*2*^ = 0.19). These indicated that responses on the left and lower sides were provided more slowly when responding to larger (vs. smaller) magnitudes. Follow-up analyses revealed that these interactions were driven by responses to larger magnitudes, which were provided about 50 ms faster with right (vs. left) and upper (vs. lower) keys (horizontal: *b* = − 0.02, *SE* = 0.01, *t* = − 2.43, *p* = 0.015; vertical: *b* = − 0.03, *SE* = 0.01, *t* = − 3.13, *p* = 0.002). In contrast, response latencies to smaller magnitudes did not differ across sides in either dimension (horizontal: *b* = 0.01, *SE* = 0.01, *t* = 1.37, *p* = 0.171; vertical: *b* < 0.01, *SE* = 0.01, *t* = − 0.23, *p* = 0.816).

Finally, a generalized logistic linear regression revealed significant accuracy decreases when responding to incongruent (left/down-large and right/up-small) response-button configurations than congruent ones (*b* = − 0.50, *SE* = 0.13, *t* = − 3.61, *p* < 0.001), and marginally more so in the vertical than in the horizontal dimension (*b* = − 0.49, *SE* = 0.27, *t* = − 1.83, *p* = 0.068). These effects validate our stimulus materials for the critical blocks.

In sum, the baseline blocks results confirmed the presence of reliable spatial–numerical associations in both the horizontal and vertical dimensions. Participants responded faster and more accurately when numerical magnitude and response side were spatially congruent, and this pattern held even when the keyboard was rotated vertically (i.e., smaller magnitudes with left and lower response buttons, and larger magnitudes with right and upper response buttons). These results show that the paradigm elicited SNARC effects across spatial axes, providing a foundation for interpreting the manipulations introduced in the subsequent critical blocks.

### Critical blocks: horizontal motion-numerical compatibility effects on magnitude classification

Table 2 reports the estimates from the linear regressions fitted to RT data from horizontal and vertical critical Motion blocks. The accuracy percentage was 98.17% (SD = 13.41%) following magnitude-congruent movement (i.e., leftward/downward-small and rightward/upward-large), and 98.23% (SD = 13.19%) following magnitude-incongruent movement. The difference between both was far from significance in both dimensions (horizontal: *p* = 0.814; vertical: *p* = 0.689).

**Table 2:** Output of mixed-effects linear regression from critical motion Blocks.

Predictor	Horizontal motion				Vertical motion			
	Est.	SE	t value	*p* value	Est.	SE	t value	*p* value
Intercept	6.67	0.05	131.95	< 0.001	6.72	0.05	134.98	< 0.001
Magnitude	− 0.01	0.04	− 0.26	0.805	− 0.04	0.03	− 1.27	0.290
Motion	< 0.01	0.01	0.01	0.996	− 0.02	0.01	− 2.84	**0.007**
Magnitude × Motion	0.03	0.01	2.32	**0.021**	< 0.01	0.01	− 0.03	0.977

Note Formula: log(RT) ~ Magnitude * Motion + (Magnitude + Motion | Participant) + (1 | Number).

Significant values are in bold.

The model fitted to data from horizontal Motion conditions revealed the hypothesized interaction of Magnitude by Motion on response times, indicating that responses given just before a rightward movement were 50 ms slower for smaller than for larger magnitudes (*f*^*2*^ = 0.20). A follow-up test confirmed that this interaction was driven by equal and opposite numerical tendencies for the effects of rightward Motion in speeding up responses to larger magnitudes by about 30 ms (*b* = − 0.01, *SE* = 0.01, *t* = − 1.42, *p* = 0.159) and slowing down responses to smaller magnitudes by about 20 ms (*b* = 0.01, *SE* = 0.01, *t* = 1.43, *p* = 0.156).

The model fitted to data from the vertical Motion condition only revealed significant effects of Motion, as participants responded more quickly when they had to turn their head upward than downward. In contrast, the Magnitude × Motion interaction was not significant in the vertical dimension. We discuss the implications of our findings in the following section.

## Discussion

The current study investigated whether motion-numerical compatibility affects the judgment of numerical magnitudes with a Go/No-go task. There are two main results: First, we successfully replicated prior evidence for both horizontal and vertical associations of smaller magnitudes with left and lower response buttons, and larger magnitudes with right and upper response buttons, using spoken numbers. Second, we found first evidence for the effect of horizontal (but not vertical) head motion on the latencies of magnitude judgments, as smaller magnitudes were judged faster when moving right-to-left, and larger magnitudes were judged faster when moving left-to-right. We discuss both results in turn.

Considering first our replication of the classic SNARC effect, we established the appropriateness of our sample size to obtain adequate statistical power, as well as the ability of our setting to detect latency signatures of space-number mappings. This result also confirms the well-documented association of physical-space and number domains, showing that response-button configurations compatible with left-to-right and bottom-to-top numerical mappings speed up latencies of magnitude judgments.

Finding standard SNARC signatures in both horizontal and vertical dimensions corroborates the view that vertical and horizontal spatial mappings co-exist and may be flexibly evoked by task settings, such as (horizontal/vertical) response-button configurations^7,9,42^. Furthermore, while SNARC effects are widely attested using visual number stimuli, such as Arabic numerals and dot patterns, the auditory modality has been studied less often. Our results replicate the available evidence for SNARC effects on latencies of keypress responses to spoken numbers^9,41^. In addition, evidence showing SNARC effects on response accuracy has been mixed, with some studies finding accuracy increases with congruent (vs. incongruent) response-button configurations^43,44^, and other studies reporting no accuracy differences (e.g^45^). Our results thus add to the available evidence supporting the role of congruency of SNAs and response-button configurations in determining accuracy of magnitude classification.

Turning now to our second result and the main research question, we had hypothesized that directional head movement may activate, via attentional deployment, selective representations of those magnitudes congruent with the movement direction, speeding up the latency of their judgment relative to a fixed reference. Such an MNC effect was thus expected to facilitate judgments of larger magnitudes when moving rightward and upward. Conversely, judging smaller magnitudes was expected to be faster when moving leftward and downward. This hypothesis was borne out only in the horizontal dimension. Namely, smaller magnitudes were judged more quickly when participants had to move leftward, and conversely larger magnitudes were judged more quickly with rightward head motion.

This novel finding indicates that directional motion toward magnitude-congruent areas of horizontal space may activate (and thus speed up the judgment of) magnitudes represented in overlapping mental space. Our observations closely align with prior evidence supporting MNC effects along horizontal space^35,36^ and extend it to a receptive magnitude-processing task, namely magnitude classification. Together, this evidence suggests that the mechanism linking physical motion and numerical processing may be mediated by visuospatial attention.

Surprisingly, however, we failed to detect an MNC effect in the vertical dimension. To the best of our knowledge, no prior experimental evidence has reported MNC effects in the vertical dimension, leaving the existence of this effect an empirical question. Yet, given the extensive evidence for SNAs in both horizontal and vertical dimensions, it seems reasonable to expect an analogous MNC effect in the vertical dimension. This expectation is supported by effects of vertical head movements on RNG^40^ and effects of RNG on vertical eye position^46^. Then why did we not find this effect?

First, it is possible that an effect of MNC in the vertical dimension does exist but merely went undetected in our experiment due to a Type 2 error. However, this seems unlikely given our ability to replicate established results and to detect a horizontal effect, as well as previous documentations of vertical SNAs with the Go/No-go task (e.g^17^). In contrast, our vertical results showed no numerical indication suggestive of motion-numerical compatibility effects (*p* = 0.977), while exhibiting comparable statistical noise as our horizontal-motion conditions.

Second, it is possible that prompting visuospatial attention along the vertical axis failed to activate the corresponding magnitude representations, while prompting attention along the horizontal axis succeeded. This discrepancy may be attributed to horizontal spatial-numerical mappings being more prominent than vertical ones^29^, especially given the symbolic nature of our spoken number stimuli. Vertical SNAs reflect evolutionary or “grounded” constraints of our physical world, such as the universal correlation between “more” and “up”; in contrast, horizontal SNAs are “embodied” cultural habits that reflect individual learning histories^47,48^. Culturally reinforced associations for symbolic magnitudes are predominantly horizontal—in the case of lists, the progression of numbers along the vertical axis may even be conceptualized from top to bottom—while vertical embodied experience of the metaphor “more is up” is generally limited to non-symbolic magnitudes. To address this possibility, vertical MNC effects may be investigated comparing symbolic numerals versus non-symbolic dot patches in future studies.

Beyond the symbolic versus non-symbolic distinction, the absence of a vertical effect might also stem from competing spatial conventions. In left-to-right reading cultures, the horizontal axis has a stable mapping direction, whereas the vertical axis involves conflicting tendencies: grounded cognition accounts predict a bottom-to-top mapping (“more is up”), while culturally learned scanning habits, such as reading lists or pages, typically proceed top-to-bottom (e.g.^47^). These opposing mappings may cancel each other, weakening any consistent vertical association. Moreover, vertical mappings might depend on whether numbers are processed as cardinal magnitudes (bottom–top) or as ordinal sequences (top–bottom). Future research should aim to disentangle these factors by manipulating both representational format and task demands.

The prominence of horizontally over vertically distributed allocation of attention is also compatible with the evolutionary basis of our visuospatial attention mechanisms^49^. For one, the human visual field is wider than it is high, reflecting the nature of human spatial exploration practices, which are generally bound to the horizontal dimension. Indeed, the mammalian navigation system relies heavily on bidimensional (hexagonal-grid) representations of the horizontal plane^50^, in which we move leftward, rightward, forward, and backwards—with little need to encode movement along the vertical axis (but see^51^, for 3D grid-cell representations in bats).

These considerations, as well as our results at large, are in line with the view that the brain circuitry enabling mammalian navigation in physical space has also been implicated in the metaphorical “navigation” of conceptual spaces^1,21^—that is, low-dimensional models in which structural relations across items are represented in terms of schematic spatial representations. Specifically, the finding that physical movement of the body shifts attention toward numerical concepts and determines their cognitive availability across tasks supports the view that physical space and mental space are isomorphic to each other^19^, and that magnitude concepts may be activated when visuospatial attention is allocated to areas of space where these concepts are represented.

Another factor that may contribute to differences between horizontal and vertical MNC effects concerns the biomechanics and motor control of head movements. Horizontal and vertical head movements involve different muscles and joint mechanics, which can influence movement fluency and ease of execution. Horizontal head rotations are typically more practiced and mechanically efficient due to frequent engagement in natural behaviors such as scanning the environment, reading, or interacting socially^52^. In contrast, vertical head movements are generally less practiced and may be more effortful or constrained by gravitational forces, potentially reducing the automaticity of attentional shifts induced by motion. These biomechanical and motoric differences could contribute to the stronger and more reliable MNC effects observed along the horizontal axis relative to the vertical axis, alongside the cognitive and cultural factors discussed above.

Additionally, participants were tested in their second language, English. While it is well known that processing mathematical content in a second language can sometimes interfere with performance^53^, participants in the present study all reported a B2 level or higher, ensuring a sufficient proficiency to understand the stimuli. The numerical stimuli were very simple (1, 2, 8, and 9), and only minimal judgments were required (smaller or larger than 5), so no complex computation was involved. Furthermore, all instructions were carefully explained and confirmed to be understood before the task. Overall, while subtle effects of second-language processing cannot be completely ruled out, the simplicity of the stimuli and our thorough instruction procedure make it unlikely that language comprehension substantially influenced the observed spatial-numerical associations. Future studies could formally assess language proficiency to examine whether it interacts with horizontal or vertical number-space mappings.

A further limitation concerns the response mapping used in the vertical condition. Participants always responded with the left hand on the lower key and the right hand on the upper key. In fact, there is evidence that when hand-to-button assignments in vertically aligned setups are held constant, anatomical or hand-based associations can dominate over genuine vertical number–space mappings^54^. Therefore, the absence of a clear vertical modulation in our critical blocks may partly reflect the stable hand configuration, which could have encouraged a response pattern grounded in motor or anatomical compatibility rather than in a conceptual up–down number mapping.

Finally, the lack of a vertical modulation may be due to our lack of statistical power to possibly detect smaller-sized effects in the vertical dimension. This is because our simulations of statistical power assumed similar size effects in both baseline and critical blocks. However, given our ability to detect an effect in the critical horizontal blocks, while our results do not exclude that an effect in the vertical dimension may in fact exist, they may be taken to indicate that motion-numerical-compatibility may be weaker in the vertical dimension.

Our study contributes to debates on the embodied and grounded nature of number representations. By demonstrating that active body movements can modulate numerical processing even in the absence of spatially coded responses, we highlight how sensorimotor engagement dynamically shapes the accessibility of abstract concepts such as numerical magnitude. Further, although head movements were bi-directional, in the critical blocks, reaction times were measured in a unimanual go/no-go task rather than a classical SNARC paradigm contrasting two response poles, and thus our results do not support polarity correspondence effects. These findings integrate spatial-numerical associations, visuospatial attention, and motor planning, highlighting the role of bodily experience in shaping number knowledge.

In addition, our results provide empirical support for the hypothesis that mental space is isomorphic to physical space, with spatial relations among cognitive representations mirroring the organization of external environments. Specifically, the modulation of numerical judgment latencies by horizontal head movements suggests that planned directional movements can bias attention toward corresponding regions of mental space, facilitating the processing of magnitude-congruent numbers. This aligns with the view that number concepts are organized along an internal mental number line and that spatial attention acts as a mechanism to access these representations. In contrast, the absence of vertical MNC effects highlights potential asymmetries in how mental space is deployed along different axes, possibly reflecting conceptual or motoric asymmetries. Overall, these findings strengthen the account that abstract numerical knowledge is grounded in spatial and attentional processes, supporting the broader notion of navigation through conceptual space.

To summarize, our findings closely align with previous evidence supporting the mapping of conceptual (especially numerical) knowledge onto horizontal and vertical space. Further, the results replicate the available evidence for motion-numerical compatibility effects in the horizontal dimension and extend it to a magnitude-classification task. Together, this evidence points to bidirectional relationships linking visuospatial attention, motor behavior, and higher cognition.

## Methods

### Participants

The experiment was approved by the Ethics Committee of the Potsdam University and conducted in accordance with the Declaration of Helsinki. The study was not pre-registered. Thirty-four participants were recruited from the participant pool of the Potsdam University and compensated with university credit for their participation.

The sample size was determined based on prospective estimations with GPower software (GPower 3.1, Universität Düsseldorf: Psychologie—HHU, Düsseldorf, Germany). A sample of 33 participants obtains power 1-*β* = 0.80 with linear regression including two predictors, assuming a medium effect size (*f*^*2*^ = 0.2) with a conventional significance criterion at *α* = 0.05.

Participants consented to participate in the study and were informed that their consent could be revoked at any time. Data from one participant was excluded due to self-reported attention-deficit disorder. The remaining 33 participants (mean age = 24 years, SD = 4 years; 25 females, 7 males, 1 non-binary) reported no history of neurological, motor, or learning impairments and were mostly right-handed (on the Edinburgh Handedness scale^55^ from − 100 to 100 from left-handers to right-handers, the mean handedness score in our sample was 86, SD = 34). 14 participants reported an English proficiency level corresponding to B2 on the Common European Frame of Reference scale (defined in the questionnaire as “You can have simple conversations on a wide range of topics”), and the remaining 19 participants reported a C1 level (“You can have extended and articulate conversation”).

### Materials

The spoken stimuli consisted of four spoken numbers in English, chosen to be either smaller than five (“one”, “two”; magnitude: small) or larger than five (“eight”, “nine”; magnitude: large). The stimuli were created through a freely available text-to-speech engine in British English spoken by a female voice (“Amy”, https://ttsmp3.com/). They were administered auditorily to participants through an over-ear headset (Sennheiser PC8). The average duration of the stimuli was 426ms (SD = 58ms). The task was scripted on OpenSesame^56^ and administered on a Dell U2715Hc laptop running Windows 11 Pro.

### Task and procedure

Upon arrival at the laboratory, participants gave their informed consent and then completed a brief questionnaire regarding their handedness^55^, English language proficiency (as both instructions and stimuli were presented in English), and any (history of) neurological, motor, or hearing impairments.

For the magnitude-classification tasks, participants sat approximately at arm’s length from a computer screen used to present instructions. Auditory stimuli consisted of the numbers 1, 2, 8, and 9, which were presented auditorily in fully randomized order.

The Experiment consisted of eight successive blocks of trials. In blocks 1 to 4 (Baseline “SNARC” blocks, Fig. 1, top panels), participants performed a magnitude-classification task by pressing one of two horizontally or vertically lateralized buttons (corresponding to letters “P” and “Q” on a QWERTZ keyboard; the “P” key was always to be responded with the right hand, while the “Q” with the left). In two of the baseline SNARC blocks, the response-key mappings were congruent with the magnitude’s position on the MNL (i.e., left/bottom key to indicate that the current number is smaller than five, and right/top key to indicate that it is larger than five). In the vertical blocks, the keyboard was continuously held by the participant in an upright orientation, with the short side laying on the table, in front of and facing away from the participant. Participants rested their fingers on the P/Q response keys—similar to the hand posture when playing a flute—and their elbows on the table. This configuration allows us to collect responses in a truly vertical (rather than e.g., sagittal) dimension. In the remaining two baseline SNARC blocks, the response-key mapping was incongruent with the MNL: participants had to press the left/bottom key to indicate that a number was larger than five, and the right/top key to indicate that a number was smaller than five. Each baseline SNARC block consisted of 60 trials. Response rules and response mappings were counterbalanced across participants.

In blocks 5 to 8 (“Critical motion blocks”, Fig. 1, lower panels), participants performed a Go/No-go magnitude-classification task on the spoken numbers by pressing the spacebar with the index finger of their dominant hand while performing repetitive horizontal or vertical head movements. Thus, each manual response directly preceded either a leftward or a rightward head movement in the horizontal dimension, and either a downward or upward movement in the vertical dimension.

Participants started each Motion block with their head turned to one side. A fixation dot was displayed for 1200 ms, after which it disappeared, and the spoken number was presented. Participants responded by pressing the spacebar if the number met the current rule and otherwise refrained from responding. After providing their response or deciding that the number did not meet the current rule and thus did not require a response, participants turned their head from the current side to the other side, without pausing at center. At the beginning of the following trial, the fixation dot presented for 1200 ms functioned as a temporal buffer (without which the next stimulus number would have been presented immediately after the Go-response), allowing participants enough time to perform the planned head turn before the next trial began. Then, the next spoken number was presented. Thus, the sequence of events within each trial was: plan head movement—hear *number—make manual response or refrain—make overt head movement*. To ensure correct execution of the head-movement responses, the experimenter stood behind the participant to monitor performance throughout the task. Importantly, no video recordings were collected at any point. This choice was made to protect participants’ identity as our ethical approval did not permit the acquisition of audiovisual data.

In two of the four critical Motion blocks, participants were asked to respond when the number was smaller than five (and to refrain from responding if it was larger than five), whereas in the other two Motion blocks, participants were asked to respond when the number was larger than five. Response rules were counterbalanced across participants. Participants completed 120 trials in each critical Motion block (60 “Go” and 60 “No-go” trials).

Before all blocks, six additional trials were presented as practice rounds. In total, each participant saw 768 trials. Participants were allowed to take brief breaks to rest between blocks. The total duration of the task was about 45 min.

### Data analysis

Data from 33 participants was included in the analysis. Due to a technical error, the first two participants did not complete all blocks. For the analyses, we selected correctly answered Go trials from the Go/No-go tasks (critical Motion blocks) and correctly answered trials from the baseline SNARC blocks (*n* = 13,536; 95.13% of the data). 26 additional trials were removed because RTs were above or below 4 SDs from the by-Participant, by-Block average (*n* = 13,510).

All analyses were performed in R Studio^57^. Data were plotted with the *ggplot2* package^58^ and analyzed with mixed-effect linear models using *lme4* and *lmerTest* packages^59,60^.

Response times (RTs) were natural-log transformed to satisfy the assumptions of our statistical approach. Magnitude (2 levels: smaller, larger) was included as within-participant categorical fixed factors in models. Response Side (in baseline SNARC blocks) and Motion (in critical Motion blocks) were treated as within-participant categorical fixed factors, each with two levels which varied across blocks (Response Side: horizontal blocks: left, right; vertical blocks: bottom, top; Motion: horizontal blocks: leftward, rightward; vertical blocks: downward, upward).

Both categorical predictors included in each model were sum-coded to compare RTs and accuracy in response to smaller magnitudes (coded as + 0.5) to the mean dependent variable across all observations (intercept). Response Side and Motion hypothesized to be congruent with larger magnitudes—that is, right and top response sides, as well as rightward and upward motion—were coded as + 0.5 and compared to the grand mean.

Random-effects structures of all models included by-Participant and by-Number intercept adjustments and by-Participant random slopes for the effects of Magnitude. In Baseline blocks, by-Participant slope adjustments for the effect of Response Side resulted in convergence errors and were thus avoided. The best-fitting random-effects structure was selected based on the lowest Akaike Information Criterion, and the results were not changed substantially by changes in the random-effects structure.

## Data Availability

All data connected to this study is available on OSF (https://osf.io/4wqg8/).
